# Identification of a common ice nucleus on hydrophilic and hydrophobic close-packed metal surfaces

**DOI:** 10.1038/s41467-023-41436-x

**Published:** 2023-09-19

**Authors:** Pengcheng Chen, Qiuhao Xu, Zijing Ding, Qing Chen, Jiyu Xu, Zhihai Cheng, Xiaohui Qiu, Bingkai Yuan, Sheng Meng, Nan Yao

**Affiliations:** 1 Princeton Materials Institute, Princeton University, Princeton, NJ 08540-8211 USA; 2 Beijing National Laboratory for Condensed Matter Physics and Institute of Physics, Chinese Academy of Sciences, 100190 Beijing, PR China; 3 Department of Physics and Beijing Key Laboratory of Optoelectronic Functional Materials & Micro-nano Devices, Renmin University of China, 100872 Beijing, PR China; 4 CAS Key Laboratory of Standardization and Measurement for Nanotechnology, CAS Center for Excellence in Nanoscience, National Center for Nanoscience and Technology, 100190 Beijing, PR China; 5 University of Chinese Academy of Sciences, 100049 Beijing, PR China; 6 Suzhou Institute of Nano-Tech and Nano-Bionics, Chinese Academy of Sciences (CAS), Suzhou, 215123 PR China

**Keywords:** Physical chemistry, Nanoscale materials, Nanoscale materials

## Abstract

Establishing a general model of heterogeneous ice nucleation has long been challenging because of the surface water structures found on different substrates. Identifying common water clusters, regardless of the underlying substrate, is one of the key steps toward solving this problem. Here, we demonstrate the presence of a common water cluster found on both hydrophilic Pt(111) and hydrophobic Cu(111) surfaces using scanning tunneling microscopy and non-contact atomic force microscopy. Water molecules self-assemble into a structure with a central flat-lying hexagon and three fused pentagonal rings, forming a cluster consisting of 15 individual water molecules. This cluster serves as a critical nucleus during ice nucleation on both surfaces: ice growth beyond this cluster bifurcates to form two-dimensional (three-dimensional) layers on hydrophilic (hydrophobic) surfaces. Our results reveal the inherent similarity and distinction at the initial stage of ice growth on hydrophilic and hydrophobic close-packed metal surfaces; thus, these observations provide initial evidence toward a general model for water-substrate interaction.

## Abstract (web summary)

One key challenge in water science is to create a universal model that can predict the arrangement of water/solid interfaces. Here, the authors use STM and noncontact-AFM to discover common water clusters during the initial growth on hydrophilic and hydrophobic close-packed metal surfaces.

## Introduction

Water/solid interfaces are critical to an incredible range of everyday phenomena and technological developments^1–4^. One of the most fundamental issues of water science is developing a general model that enables the prediction of the atomic structure and dynamics of water/solid interfaces. Identifying water structures with atomic resolution, especially common stable water clusters, regardless of the substrate, can provide direct evidence for the development of a general model of water adsorption and facilitate a deeper understanding of water-substrate interactions.

Since the 1980s, the conventional hexagonal bilayer model^5,6^ based on the Bernal–Fowler–Pauling ice rules, which consists of a buckled hexagonal overlayer with water molecules at two distinct heights, has been widely used as the “standard model” to describe water structures at various interfaces. However, molecular-level studies have shown that a diversity of surface water structures^7–15^ forms on surfaces, and their atomic structures vary from substrate to substrate. The rich variety of water structures on surfaces, ranging from isolated clusters^7,10,11,16–18^, quasi-one-dimensional chains^8,19^, two-dimensional (2D) wetting layers^12,15,20,21^ to three-dimensional (3D) islands^22^, challenge the validity of the standard bilayer model^6^ and imply that there is no general model that can accurately describe water structures on surfaces. Recently, a set of “2D ice rules”^16^ has been proposed based on the observation of the same bonding motif featuring flat-lying hexagonal rings bridged by pentagonal and heptagonal rings on several hydrophilic metal surfaces at low temperatures^9,15,16,20,23^. However, it is difficult to extend this set of rules to interpret distinct water structures observed on hydrophobic surfaces in previous reports^7,17^. One type of 2D bilayer hexagonal ice and its derivative are observed on several hydrophobic surfaces^12,24–26^, revealing the generality of this structure. In contrast, diverse structures are observed on hydrophilic surfaces. Hence, there is a great gap between the water structures on hydrophilic and hydrophobic metal surfaces, and common water clusters on both types of surfaces have not been identified. In addition, early molecular-level studies have not been free from the controversy over the detailed water structure assignments, and similar water clusters on various metals could be identified as different structures. This controversy may result in overlooking the hidden similarity of water structures on different substrates.

Recent developments in noncontact atomic force microscopy (NC-AFM) enable real-space characterization of the atomic structure of surface water with precise positional information of the oxygen atoms^12–14,23,25,26^ along with corresponding O-H bond orientations^27,28^. Here, we use this method to image the structure of a critical ice nucleus formed on hydrophilic Pt(111) and hydrophobic Cu(111) surfaces at the atomic scale and have revealed the inherent similarity in ice nucleation on both surfaces.

## Results

### Resolves the structure of water clusters

**Fig. 1:**
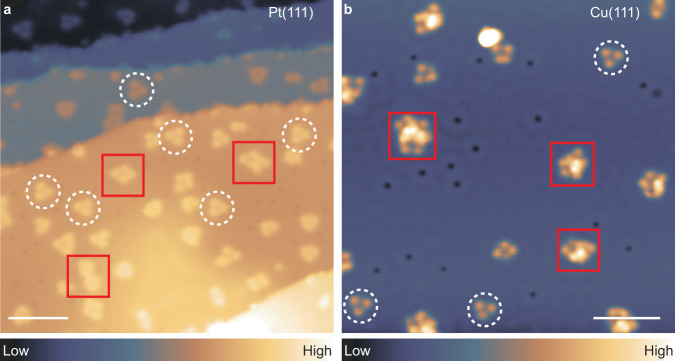
STM images of adsorbed water clusters on Pt(111) and Cu(111) with a CO-terminated tip. **a** Constant-current STM image of water clusters on Pt(111) (*V* = 100 mV, *I* = 30 pA). **b** Constant-current STM image of water clusters on Cu(111) (*V* = 100 mV, *I* = 30 pA). The water clusters with threefold symmetry are marked with white dashed cycles. Larger clusters on both surfaces are marked with red rectangles. Scale bars: 5 nm.

Sample preparation for both surfaces was with identical parameters. Water molecules were deposited on Pt(111) and Cu(111) surfaces at 4.8 K. After annealing the substrate above 25 K, various water clusters were formed. Figure 1 shows representative STM images of water clusters on both surfaces. These water clusters are isolated on the terraces and have several bright lobes. We observed a popular existence of water clusters with a threefold symmetry on both surfaces, as marked by white dashed circles in Fig. 1a, b. It can also be seen that larger clusters show a flat and relatively ordered structure on the Pt(111) surface, while more amorphous clusters are observed on the Cu(111) surface, as marked by red boxes in Fig. 1.

**Fig. 2:**
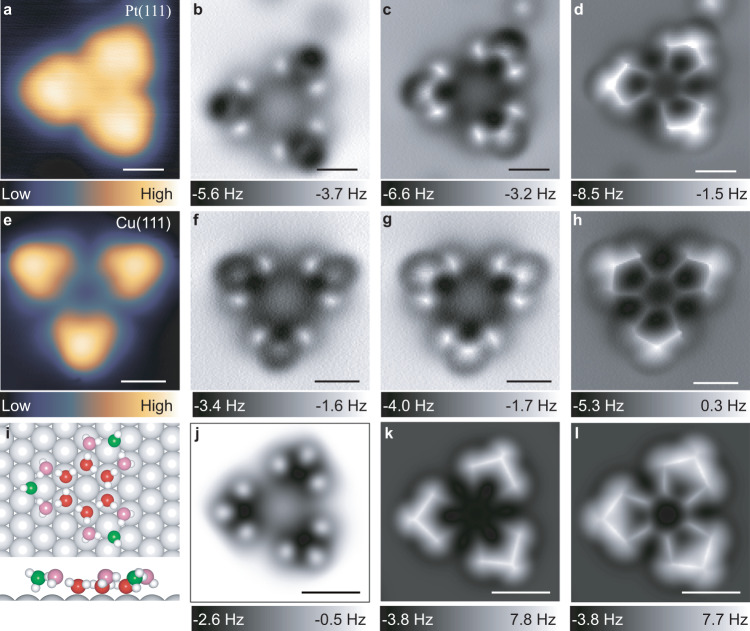
SPM characterization of water clusters with threefold symmetry on Pt(111) and Cu(111) with a CO-terminated tip and the corresponding structural models. **a**, **e** Constant-current STM images (*V* = 100 mV, *I* = 30 pA) of water clusters on Pt(111) and Cu(111), respectively. Constant-height AFM (Δ*f*) images of water cluster with threefold symmetry on the Pt(111) surface at tip heights of +30 pm (**b**), +10 pm (**c**), and −20 pm (**d**). Constant-height AFM (Δ*f*) images of water cluster with threefold symmetry on the Cu(111) surface at tip heights of +30 pm (**f**), +10 pm (**g**), and −40 pm (**h**). **i** Top and side views of the water cluster structure on Pt(111). The water cluster on the Cu(111) surface has a similar structure. H atoms are denoted as small white spheres, Pt atoms are denoted as large-width spheres, and O atoms in low, high, and H-down water molecules are denoted as red, pink, and green spheres, respectively. The tip heights in **b**–**d** and **f**–**h** are referenced to the STM setpoint (*V* = 100 mV, *I* = 30 pA) on the Pt(111) and Cu(111) surfaces, respectively. Simulated AFM images based on the structural model on Pt(111) at tip heights of 15.7 Å (**j**), 15.4 Å (**k**), and 14.8 Å (**l**). Scale bars: 0.5 nm.

We first discuss the water clusters that possess a threefold symmetry observed on both surfaces shown in Fig. 1. Figure 2b–d shows the constant-height frequency shift (Δ*f*) NC-AFM images of the water cluster on Pt(111) acquired at different tip heights when using a CO-terminated tip. In contrast to the STM image (Fig. 2a), the NC-AFM images (Fig. 2b–d) revealed an atomically resolved structure. At a larger tip height, six bright protrusions and three depressions are imaged at the three protrusions in the STM image (Fig. 2b). At a closer tip height, the initial three depressions at the outer end appear as bright protrusions, and nine protrusions are observed with bonding-like features between the protrusions (Fig. 2c). The contrast in NC-AFM images mainly arises from Pauli repulsion at close tip-sample distance^29,30^ which increases with the electron density. As revealed by previous studies^13^, the bright protrusions in NC-AFM images show the lateral position of oxygen atoms of water molecules. Hence, the observed protrusions suggest that nine water molecules are visible, and the contrast inversion indicates that these molecules can be classified as two types of water species with different adsorption heights and orientations. When the tip height decreases further, the six water molecules at the center become visible (Fig. 2d). Thus, the water cluster with threefold symmetry has 15 water molecules (Fig. 2i) connected by hydrogen bonds (H-bond), which consists of a central hexagonal ring and three peripheral pentagonal rings with three types of water molecules involved. This unique cluster is denoted as the 15-mer hereafter. The structure assignment of the 15-mers was further proven by our simulated NC-AFM images (Fig. 2j–l), which agree well with the experimental results. It is noticed that H-bond orientations in water clusters can be discriminated by STM^11^ and NC-AFM^12,28^ in previous studies. However, the H-bond orientation is not discriminable here, as the H atoms are almost invisible from the AFM images. In fact, the energy of these flat 15-mer structures with clockwise or counterclockwise H-bond orientations degenerates according to ab-initio calculations, so they should appear with equal possibilities.

A similar contrast is observed for the water cluster grown on the Cu(111) surface, as shown in Fig. 2f–h. This clearly indicates that the water clusters with threefold symmetry adopt the same structure on the Pt(111) and Cu(111) surfaces. We note that the STM images of 15-mers resemble the water clusters with threefold symmetry grown on Cu(111) and Ag(111) surfaces in previous STM studies. They were previously proposed either as a nonamer comprising a central hexamer with three additional water molecules^7,18,31^ or an 18-mer comprising four hexamers^17^. The comparison of lateral sizes of these water clusters from previous studies and our study with threefold symmetry are summarized in Supplementary Fig. 1.

It is important to determine the adsorption sites of each water molecule in the 15-mer to discuss the stability of the cluster grown on different substrates. It is known that CO binds to the top site on Pt(111)^32^ and Cu(111)^33^ with an upright configuration. With the help of CO and atomic images of the substrates, we can determine the adsorption sites of the water molecules in the 15-mer. As shown in Supplementary Fig. 2, the O atoms of the central hexagon ring adsorb on the top sites of Pt(111) but shift to the inner side to form short H-bonds. The six molecules bonded to the hexagon ring also adsorb around the top sites, while the three water molecules with contrast inversion adsorb near the hollow sites. Compared to that on Pt(111), the adsorption sites of the water molecules in the 15-mer on Cu(111) are similar but show the influence of a smaller lattice constant (Supplementary Fig. 2). The same behavior also appears in the atomic structures optimized by density functional theory (DFT) (Supplementary Fig. 3).

### Structure determination of water clusters with DFT

**Fig. 3:**
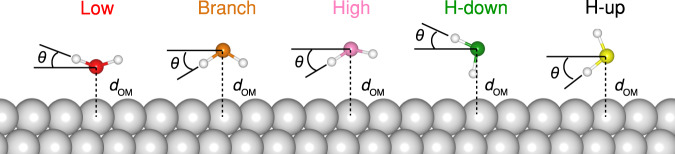
Molecular adsorption configurations in water clusters on Pt(111) and Cu(111) obtained from DFT calculations. The H atoms are denoted as white spheres. The O atoms in Low, Branch, High, H-down, and H-up water molecules are denoted as red, brown, pink, green, and yellow spheres, respectively.

Based on the structural information obtained from the NC-AFM images, DFT calculations were performed to elucidate further the 15-mer water cluster observed here. Optimization of atomic structures from DFT confirms that the topological structure of these 15-mers is identical on both Pt(111) and Cu(111) surfaces. Water molecules included within these clusters can be classified into three types based on their heights and orientations (Fig. 2i). Those molecules in the central hexagon are flat-lying and strongly bound onto the top site of the Pt or Cu substrates (denoted as low-water in Table 1 and Fig. 3). The water-Cu distance is approximately 10 pm larger than that of water-Pt, indicating a stronger interaction between water and Pt. Each low-water donates two H-bonds and accepts one H-bond. The six water molecules bonded to this low-water (hexagon ring) also adopt a flat configuration but have a larger distance relative to the substrate (denoted as high-water in Table 1 and Fig. 3). It is noted that experimentally confirmed adsorption sites shift slightly from the center of top sites. The water molecules (denoted as H-down in Table 1 and Fig. 3) at the end of the peripheral pentagons adopt a vertical geometry with one OH pointing down to the hollow site of the surface and one OH pointing up with an angle, which can well explain the contrast inversion of NC-AFM images in Fig. 2b, c, f, g. At large tip-sample distances, the attractive interaction between the CO tip and the dangling -OH pointing up^25^ results in the observed depressions. In contrast, Pauli repulsive interaction dominates at close tip-sample distances. Overall, the mixture of 5- and 6-membered rings of water molecules is stable in energy because a significant fraction of them lie flat and close to the substrates, and they complete a closed-loop structure simultaneously, which maximizes their water–metal interaction and the number of H-bonds.

**Table 1:** Molecular adsorption configurations in water clusters on Pt(111) and Cu(111) obtained from DFT calculations

	*N*_A_	*N*_D_	Sites	*E*_water-Pt/Cu_	*d*_OM_(Å)	*θ* (°)
Low	0 1	0 – 2 1, 2	Top	Strong	2.2–2.3	<30
Branch^*^	1	0	All	Weak	≈3.2	>−30
High	1	1, 2	All	Medium	3.2–3.4	<−30
H-down	2	0	Hollow	Weak	≈3.5	≈30
H-up	2	1	Top	Weak	≈3.5	≈−30

*N*_A_ and *N*_D_ are the number of acceptors and donors water serves, respectively. *E*_water-Pt/Cu_ is the water-Pt(111) and water-Cu(111) interaction strength, *d*_OM_ is the O-Pt and O-Cu bond length, and *θ* is the polar angle water makes with the surface plane.

^*^Water is not a component of molecular rings.

### The similarity of water clusters on different surfaces

It has been reported that the first water-wetting layer on Pt(111) is built with flat-lying central hexagonal rings surrounded by three alternately fused pentagonal and three heptagonal rings^15,20^. These studies suggest that part of the building unit on Pt(111) is made of the same structure motif as what we observed in the 15-mer, including the flat structure of the central hexagonal ring, along with the height and -OH orientation of the water molecules in the peripheral pentagonal rings. The similar calculations^15,20^ provided by these authors complement our own study and show that the film built by repeating this unit is lower in energy than any competing hexagonal water network. The energy gain is also similar to that of a 15-mer on Pt(111) and Cu(111) surfaces in our experiments. A bonding motif similar to the 15-mer has also been observed on several hydrophilic close-packed metals surfaces^14,20,21,34,35^, such as Pd(111), Ru(0001), and Ni(111) (Supplementary Fig. 4). A similar water-wetting layer is observed on Ni(111)^14,34^, while the 15-mer is observed at the boundary of the water network on Pd(111) and Ru(0001)^35^. We further calculate the adsorption energies of 14, 15, 16-mers on these metal substrates (Supplementary Table 1). DFT calculations show that the adsorption energy of the 15-mers is larger than 14-mers or 16-mers except for clusters on Ag substrates, which means the 15-mers are stable energetically. These complementary studies suggest that the 15-mer motif is a stable structure motif on various hydrophilic close-packed metal surfaces with strong water-metal interaction, despite the broad lattice mismatch between bulk ice film and these metal substrates^5^.

The observation of 15-mers on hydrophobic Cu(111) surfaces was unexpected, considering the weaker water-metal interaction and the distinct structures proposed in previous studies^7,17,18,30^. Nevertheless, observing the 15-mer on various hydrophilic and hydrophobic close-packed metal surfaces demonstrates its important role in heterogeneous ice nucleation at the initial stage, which is crucial for developing a generic model but has been overlooked before.

15-mer derivatives with similar water clusters were also observed on non-hexagonal metal surfaces, while their exact geometry may vary and adapt to the symmetry of the new substrate. We studied non-hexagonal metal Cu(110) surfaces, and water clusters with one central hexagon and several fused pentagons were observed, as shown in Supplementary Fig. 5. Due to the different lattice symmetries, water clusters with twofold symmetry are mainly formed, revealing the influence of substrate symmetry. The observation of 15-mer derivatives on Cu(110) indicates that these structures may be formed on more metal surfaces.

### The growth process of the water clusters

**Fig. 4:**
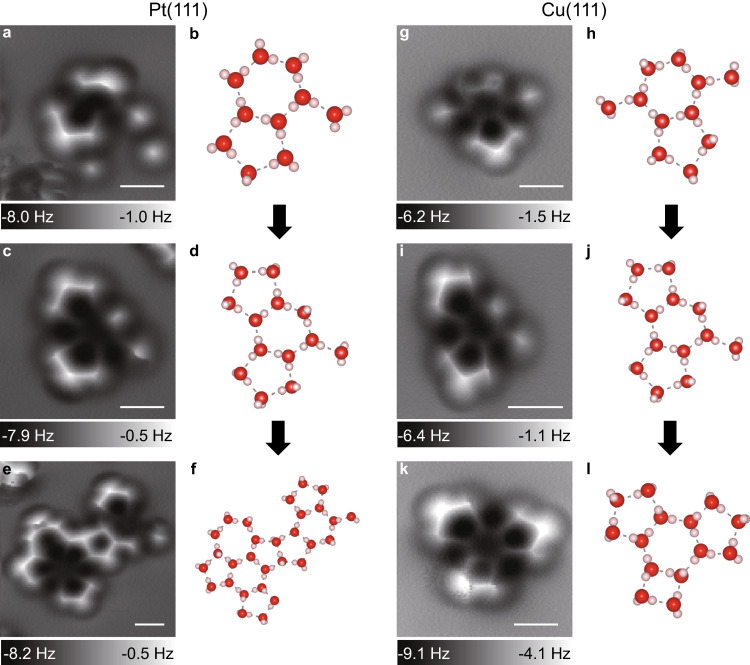
AFM characterization of intermediate water clusters on Pt(111) and Cu(111) surfaces with a CO-terminated tip. **a**, **c**, **e** Constant-height AFM (Δ*f*) images of water clusters on Pt(111). **b**, **d**, **f** The corresponding ball-and-stick models of the water clusters on Pt(111). **g**, **i**, **k** Constant-height AFM (Δ*f*) images of water clusters on Cu(111). **h**, **j**, **l** The corresponding ball-and-stick models of the water clusters on Cu(111). H and O atoms in **g**, **i**, **k**, **h**, **j**, and **l**, are denoted as pink and red spheres, respectively. The dashed lines in **b**, **d**, **f**, **h**, **j**, and **l** indicate the H bonds between water molecules. Scale bars: 0.5 nm.

Revealing the growth process of the 15-mer clusters can further clarify their roles in the initial stage of water cluster growth on both hydrophilic and hydrophobic metal surfaces. Several intermediate clusters are characterized using NC-AFM. Similar intermediate clusters are observed on both surfaces. Figure 3a, b shows a water cluster on Pt(111) consisting of a hexagon and a pentagon with an additional molecule bonded on the hexagonal ring. A similar water cluster is observed on Cu(111). The water cluster in Fig. 3g, h contains a hexagon and a pentagon with two additional molecules bonded at the para-positions. For stable clusters such as 15-mer, we don’t find observer notable perturbations for the water molecules during imaging. However, for clusters with unstable dangling water molecules, such as Fig. 4g, the affecting of the water molecule from the tip can be observed. Additional water molecules can attach to different positions of the central hexagonal ring for further growth. While two additional water molecules attach to the hexagon at ortho-positions, one more tetragon is formed due to a weak H-bond between them, as shown in Fig. 4k, l. The pentagon ring is formed with one water molecule bridged between the two molecules at ortho-positions, as shown in Fig. 4c, d, i, j.

The observation of similar intermediate clusters on both Pt(111) and Cu(111) strongly suggests that the two surfaces share similar nucleation and growth processes, forming fused pentagonal and hexagonal structures. These processes include i) the formation of a hexagon ring, ii) the attachment of individual water molecules at ortho-positions along the central hexagon ring to form a tetragon, and iii) the formation of a pentagon with an additional water molecule bridged between the two molecules at the ortho-position. The pentagon rings could also be formed at the para-positions of the hexagon ring. Our theoretical simulations of the growth of 15-mer clusters on Pt and Cu corroborated these observations.

Although the hydrophobic surfaces (Cu(111)) and hydrophilic surfaces (Pt(111)) share this critical starting nuclei structure, these surfaces diverge from one another and form a unique water network at higher coverages. For Pt(111), the water cluster in Fig. 3e, f shows the formation of a heptagonal ring bridged between two pentagonal rings, which represents an intermediate from the 15-mer to the wetting layer on Pt (111)^15^ and Ni(111)^14^ at higher temperatures, which consists of repeated hexagonal, pentagonal and heptagonal rings. For larger 2D water clusters on Pt(111), a 2D growth mode is favored (Supplementary Fig. 6), indicating the wetting behavior of water on Pt(111). In contrast, these kinds of 2D intermediates cannot form on Cu(111). Instead of forming heptagonal and pentagonal rings, the clusters with several more water molecules tend to form more hexagonal rings, as shown in Supplementary Fig. 6. For larger water clusters, a 3D growth mode is favored on Cu(111), and this first layer consists of hexagonal rings with pentagonal rings at the edge (Supplementary Fig. 6). This is supported by the large amorphous clusters marked by red boxes in Fig. 1b and is consistent with the formation of large 3D clusters and islands on hydrophobic surfaces in previous studies (Supplementary Fig. 7)^22^.

After comparing the nucleation and growth process on Pt(111) and Cu(111), we believe that the 15-mer motif serves as a critical nucleus that is necessary for ice nucleation on both surfaces: water molecules form a 15-mer first, and ice growth beyond this cluster bifurcates to form two-dimensional (three-dimensional) layers on hydrophilic (hydrophobic) surfaces.

**Fig. 5:**
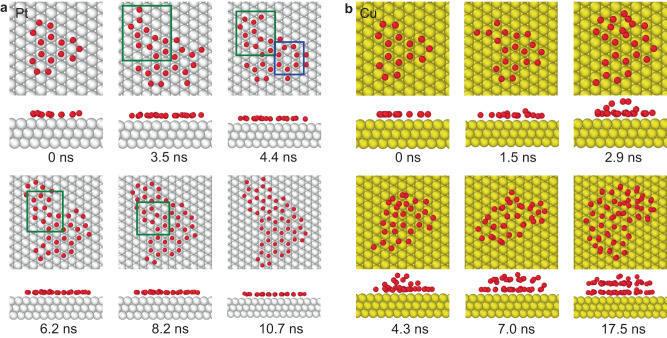
Simulated ice growth from a 15-mer to a 2D film (3D island) on wetting (nonwetting) surfaces. **a** Snapshots from the simulated ice growth trajectory on Pt(111). **b** Snapshots from the simulated ice growth trajectory on Cu(111). The green boxes indicate the formation of a chain of pentagon rings, and the blue box indicates the formation of a heptagon ring. The water molecules, Pt, and Cu atoms are denoted as red, white, and yellow spheres, respectively.

The experimental results discussed above are further confirmed by theoretical simulations of ice growth on Pt(111) and Cu(111) surfaces. There are two typical pathways to form a large planar structure from the 15-mer. One forms a chain of pentagons constructed by the original nucleus and newly deposited water molecules, followed by subsequent ring merging (Fig. 5a, green box). The other is to form the heptagonal ring directly, which remains stable until it is surrounded by other pentagons and hexagons (Fig. 5a, blue box). Eventually, water molecules form an extended overlayer on Pt(111). On the other hand, water quickly forms multilayers after forming a 15-mer on Cu(111). After adsorbing 4-5 newly deposited water molecules, the 15-mer cluster is further lifted from the surface to release the accumulated strain and eventually form multilayer structures with disordered edges and discontinuous uppermost layers (Fig. 5b). The 3D growth results from the weaker water-metal bonding strength^5,7^ on hydrophobic surfaces; this interaction is not strong enough to compete with the H-bond formed between the first layer and second layer and ultimately limits two-dimensional growth.

This study’s results help rationalize the previously observed water structures on heterogeneous surfaces and provide direct evidence of the inherent similarity of heterogeneous ice nucleation on hydrophobic Cu(111) and hydrophilic Pt(111) surfaces. By combining NC-AFM and theoretical calculations, we have identified the formation of a key 15-mer motif observed on both Pt(111) and Cu(111) surfaces. In addition, we demonstrate that the 15-mer cluster is a critical nucleus that plays a vital role during the initial stages of ice nucleation on these surfaces. This cluster and its derivatives may also exist on other metal substrates to assist in ice nucleation. Ultimately, this study facilitates the basis necessary for building a general model that defines heterogeneous ice nucleation at the atomic scale.

## Methods

### STM and AFM measurements

All the experiments were carried out in two ultra-high vacuum low-temperature scanning probe microscopes (Createc and Omicron). STM and AFM images were acquired at 4.8 K. The bias voltages referred to the sample in both STM and AFM images. The STM images were recorded in constant-current mode. The AFM images were recorded in constant-height frequency modulation mode using qPlus sensors equipped with a tungsten tip (resonance frequency *f*_0_ ≈ 30.0/26.7 kHz, bias *V* = 0 V, oscillation amplitude *A* ≈ 100 pm, the quality factor *Q* ≈ 10^4^) with the CO-terminated tip. CO molecules were in-situ evaporated onto the Pt(111), Cu(111), and Cu(110) surfaces at 4.8 K. In experiments, the tips were further cleaned and shaped by a combination of bias pulses and controlled indentation into the metal substrate. CO-terminated tips were prepared by picking up CO from Pt(111) and Cu(111) or NaCl island on Cu(110). The tip height Δ*z* was set with respect to a reference height given by the STM set point above the bare metal substrate in the vicinity of the water clusters. The plus (minus) sign means the increase (decrease) of tip height.

### Sample preparation

The Pt(111), Cu(111), Cu(110), and Ag(11) single crystals were cleaned by repeated cycles of sputtering and annealing. The water of milli-Q quality was further purified under vacuum by freeze-thaw cycles. Water was dosed onto the Pt(111) and Cu(111) surfaces through a leaking valve while keeping the sample at 4.8 K. Water is adsorbed molecularly at this temperature, and monomer diffusion is quenched. The ordered water clusters can be produced by annealing the sample above 25 K. Water was dosed onto the Cu(110) surface through a leaking valve while keeping the sample at 78 K. The sample on Cu(110) was checked by STM at 78 K and then cooled down to 4.8 K for STM and AFM measurements. Water was dosed onto the Ag(111) surface through a leaking valve while keeping the sample at 4.8 K. The sample on Ag(111) was measured by STM and AFM without annealing.

### First-principles calculations

First-principles calculations have been performed within the density functional theory (DFT) framework using the Vienna Ab-initio Simulation Package code (VASP). The geometries of water clusters have been optimized using Projector-augmented wave pseudo-potentials and van der Waals density functional (optB88-vdw). The substrate consists of five atomic layers, with lattice constants of 3.61 Å for Cu and 3.92 Å for Pt which is taken from experimental values. An (8 × 8) surface unit cell of Pt(111) and Cu(111) is constructed to hold water clusters to avoid the error from water-image interactions. The plane-wave energy cutoff is set as 520 eV for all the atoms. All atoms except those in the bottom two layers are relaxed during geometry optimization until the magnitude of forces is less than 0.05 eV Å^−1^. The Brillouin zone was sampled with 3 × 3 × 1 k points using the Monkhorst-Pack scheme.

Adsorption energy was calculated by subtracting the total energy of the adsorption system from the sum of the energies of the relaxed bare metal substrate and n isolated water molecules in gas phase:

1 $${E}_{{{{{{\rm{ads}}}}}}}=({E}_{{{{{{\rm{metal}}}}}}}+n\times {E}_{{{{{{{\rm{H}}}}}}}_{2}{{{{{\rm{O}}}}}}}-{E}_{\left({{{{{{\rm{H}}}}}}}_{2}{{{{{\rm{O}}}}}}\right){{{{{\rm{n}}}}}}/{{{{{\rm{Metal}}}}}}})/n$$

The adsorption energies of 15-mers (14-mers and 16-mers on Pt and Ag(111)) on different metal substrates are shown in Supplementary Table 1.

### AFM simulations

AFM simulations are performed by probe particle model, where three forces: 1) spring forces; 2) Pauli repulsion and van der Waals attraction between the probe particle and fixed atoms of the substrate; 3) electrostatic forces between sample and charged probe particle (Hartree potential are calculated via DFT) are considered. Codes are developed by Nanosurf Lab.

### The definition of the hydrophobicity and hydrophilicity

The concept of hydrophobicity and hydrophilicity at the single-molecule level are previously mainly characterized by the water–surface coupling and the strength of the hydrogen bond at the interface, which highlighted the importance of our work. Our results clearly revealed the hydrophobic and hydrophilic behaviors of the two surfaces at the molecular scale.

### Molecular dynamics simulations

Our Molecular Dynamics (MD) simulations were carried out using the LAMMPS package. Water-water interactions were described by the monoatomic model, which consists of short-ranged two-body and three-body non-bonding potentials without explicitly including hydrogen atoms. The 12-6 Lennard-Jones potential with a 10 Å cutoff was used to interact with water and metal atoms of Pt(111) and Cu(111) surfaces. The Lennard-Jones parameters were determined as *ε*_Pt-water_ = 0.72 kcal mol^−1^ (*ε*_Cu-water_ = 0.40 kcal mol^−1^) and *σ*_Pt-water_ = 2.82 Å (*σ*_Cu-water_ = 2.56 Å) to match the DFT result. The velocity Verlet algorithm was used to integrate the equations of the motion with a time step of 2 fs. Metal atoms were fixed during molecular-dynamics simulations, and periodic boundary conditions were applied in in-plane directions of the simulation box.

We introduce a water molecule every 0.3 ns on the Pt sheet with an area of 47.65 Å × 49.22 Å and the Cu sheet with an area of 49.06 Å × 49.17 Å to simulate the deposition process. The water molecule was initially located 15−20 Å above the metal surfaces and was given initial velocities with a random magnitude from 5.0 to 10.0 Å ps^−1^ in the direction towards the metal surface. All simulations were performed in a constant-volume and constant-temperature (NVT) ensemble. The temperature was set to 150 K and controlled using a Langevin thermostat with a relaxation time of 1 ps^−1^.

### Reporting summary

Further information on research design is available in the Nature Portfolio Reporting Summary linked to this article.

### Supplementary information

Supplementary Information

Reporting Summary

## Data Availability

The data that support the findings of this paper are available from the corresponding authors upon request.

## References

[1] Li G, Wang B, Resasco DE (2020). Water-mediated heterogeneously catalyzed reactions. ACS Catal..

[2] Björneholm O (2016). Water at interfaces. Chem. Rev..

[3] Bampoulis P, Sotthewes K, Dollekamp E, Poelsema B (2018). Water confined in two-dimensions: fundamentals and applications. Surf. Sci. Rep..

[4] Meng S, Greenlee LF, Shen YR, Wang E (2015). Basic science of water: challenges and current status towards a molecular picture. Nano Res..

[5] Hodgson A, Haq S (2009). Water adsorption and the wetting of metal surfaces. Surf. Sci. Rep..

[6] Carrasco J, Hodgson A, Michaelides A (2012). A molecular perspective of water at metal interfaces. Nat. Mater..

[7] Michaelides A, Morgenstern K (2007). Ice nanoclusters at hydrophobic metal surfaces. Nat. Mater..

[8] Carrasco J (2009). A one-dimensional ice structure built from pentagons. Nat. Mater..

[9] Tatarkhanov M (2009). Metal- and Hydrogen-bonding competition during water adsorption on Pd(111) and Ru(0001). J. Am. Chem. Soc..

[10] Chen J (2014). An unconventional bilayer ice structure on a NaCl(001) film. Nat. Commun..

[11] Guo J (2014). Real-space imaging of interfacial water with submolecular resolution. Nat. Mater..

[12] Ma R (2020). Atomic imaging of the edge structure and growth of a two-dimensional hexagonal ice. Nature.

[13] Shiotari A, Sugimoto Y (2017). Ultrahigh-resolution imaging of water networks by atomic force microscopy. Nat. Commun..

[14] Shiotari A, Sugimoto Y, Kamio H (2019). Characterization of two- and one-dimensional water networks on Ni(111) via atomic force microscopy. Phys. Rev. Mater..

[15] Nie S, Feibelman PJ, Bartelt NC, Thürmer K (2010). Pentagons and heptagons in the first water layer on Pt(111). Phys. Rev. Lett..

[16] Salmeron M (2009). Water growth on metals and oxides: binding, dissociation and role of hydroxyl groups. Faraday Discuss.

[17] Liriano ML (2017). Water–ice analogues of polycyclic aromatic hydrocarbons: water nanoclusters on Cu(111). J. Am. Chem. Soc..

[18] Mehlhorn M, Carrasco J, Michaelides A, Morgenstern K (2009). Local investigation of femtosecond laser induced dynamics of water nanoclusters on Cu(111). Phys. Rev. Lett..

[19] Gerrard N, Mistry K, Darling GR, Hodgson A (2020). Formation of linear water chains on Ni(110). J. Phys. Chem. Lett..

[20] Maier S, Lechner BAJ, Somorjai GA, Salmeron M (2016). Growth and structure of the first layers of ice on Ru(0001) and Pt(111). J. Am. Chem. Soc..

[21] Maier S, Salmeron M (2015). How does water wet a surface?. Acc. Chem. Res..

[22] Mehlhorn M, Morgenstern K (2007). Faceting during the transformation of amorphous to crystalline ice. Phys. Rev. Lett..

[23] Guo, J. et al. Probing water structure on Pt(111) with noncontact atomic force. In *22nd International Conference on Non-contact Atomic Force Microscopy*. p54 (2019).

[24] Kimmel G (2009). No confinement needed: observation of a metastable hydrophobic wetting two-layer ice on graphene. J. Am. Chem. Soc..

[25] Yang P (2022). Robustness of bilayer hexagonal ice against surface symmetry and corrugation. Phys. Rev. Lett..

[26] Tian Y (2022). Visualizing Eigen/Zundel cations and their interconversion in monolayer water on metal surfaces. Science.

[27] Kawakami N, Iwata K, Shiotari A, Sugimoto Y (2020). Intrinsic reconstruction of ice-I surfaces. Sci. Adv..

[28] Peng J (2018). Weakly perturbative imaging of interfacial water with submolecular resolution by atomic force microscopy. Nat. Commun..

[29] Gross L, Mohn F, Moll N, Liljeroth P, Meyer G (2009). The chemical structure of a molecule resolved by atomic force microscopy. Science.

[30] Gross L (2012). Bond-order discrimination by atomic force microscopy. Science.

[31] Guo Y (2016). Inducing transient charge state of a single water cluster on Cu(111) surface. ACS Nano.

[32] Kasun GT, Gunasooriya K, Saeys M (2018). CO adsorption on Pt(111): from isolated molecules to ordered high-coverage structures. ACS Catal..

[33] Heinrich AJ, Lutz CP, Gupta JA, Eigler DM (2002). Molecule cascades. Science.

[34] Thürmer K, Nie S, Feibelman PJ, Bartelt NC (2014). Clusters, molecular layers, and 3D crystals of water on Ni(111). J. Chem. Phys..

[35] Maier S (2012). Adsorbed water-molecule hexagons with unexpected rotations in islands on Ru(0001) and Pd(111). Phys. Rev. B.

