# Supplementary material for: Identification of a common ice nucleus on hydrophilic and hydrophobic close-packed metal surfaces
Source: Nat Commun. 2023 Sep 19;14:5813. doi: 10.1038/s41467-023-41436-x (PMC10509196; doi:10.1038/s41467-023-41436-x)
Supplement: Supplementary file 1 — Supplementary Information [file 41467_2023_41436_MOESM1_ESM.pdf]

Supplementary information for
Identification of a common ice nucleus on hydrophilic and hydrophobic close-packed metal surfaces

Authors: Pengcheng Chen[1]†, Qiuhao Xu[2]†, Zijing Ding[2], Qing Chen[2], Jiyu Xu[2], Zhihai Cheng[3], Xiaohui Qiu[4,5]*, Bingkai Yuan[6]*, Sheng Meng[2,5]*, and Nan Yao[1]*

Affiliations:

1) *Princeton Materials Institute, Princeton University, Princeton, New Jersey, 08540-8211, USA*

2) *Beijing National Laboratory for Condensed Matter Physics and Institute of Physics, Chinese Academy of Sciences, Beijing 100190, P. R. China.*

3) *Department of Physics and Beijing Key Laboratory of Optoelectronic Functional Materials & Micro-nano Devices, Renmin University of China, Beijing 100872, China.*

4) *CAS Key Laboratory of Standardization and Measurement for Nanotechnology, CAS Center for Excellence in Nanoscience, National Center for Nanoscience and Technology, Beijing 100190, China.*

5) *University of Chinese Academy of Sciences, Beijing 100049, China.*

6) *Suzhou Institute of Nano-Tech and Nano-Bionics, Chinese Academy of Sciences (CAS), Suzhou 215123, China.*

†*These authors contributed equally: Pengcheng Chen, Qiuhao Xu.*

**To whom correspondence should be addressed:*

nyao@princeton.edu; smeng@iphy.ac.cn; bkyuan2023@sinano.ac.cn; xhqiu@nanoctr.cn.

substrate	Pt(fcc)	Pt(hcp)	$Pt_{(14\text{-mers})}$	$Pt_{(16\text{-mers})}$	Cu	Au	Ni	Ru
E_{ads}(meV)	654.2	666.5	640.2	647.7	594.3	553.9	641.8	749.4

substrate	Ag	$Ag_{(14\text{-mers})}$	$Ag_{(16\text{-mers})}$
E_{ads}(meV)	529.3	518.0	529.8

Supplementary Table 1: The adsorption energies of 15-mers on fcc hollow site of Pt(111), hcp hollow site of Pt(111), Cu(111), Au(111), Ag(111), Ni(111), Ru(0001) surfaces and 14, 16-mers on Pt(111), Ag(111) surfaces. The top view of these clusters is shown in Supplementary Fig. 9. The adsorption energy of 14, 15, 16-mers are compared. DFT calculations show that 15-mers can also exist on other substrates. Besides, except for clusters on Ag substrates, the adsorption energy of the 15-mers is larger than 14-mers or 16-mers, which means the 15-mers is stable in energetically.

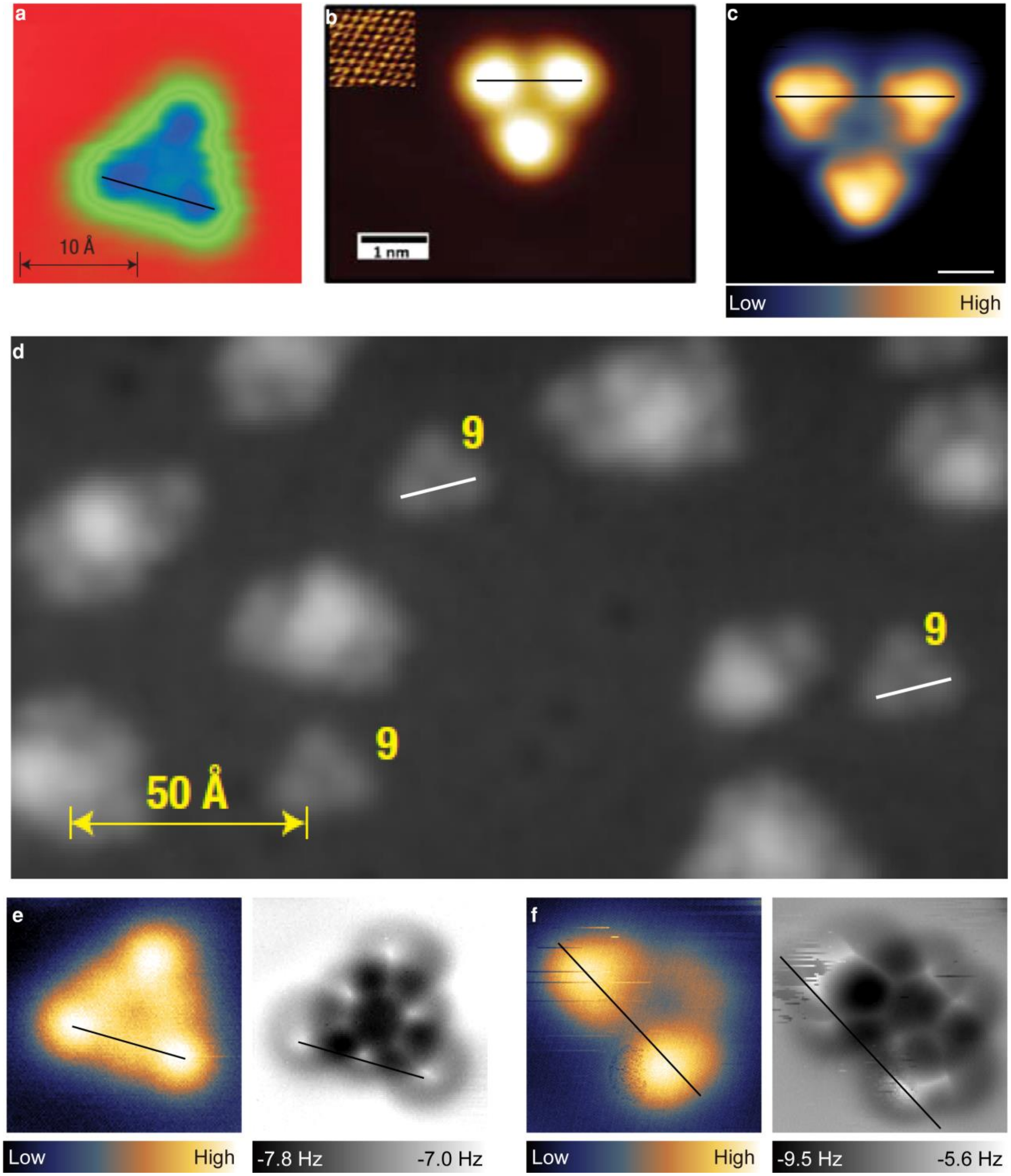

Supplementary Fig. 1 | Comparison of lateral size of ice clusters on Ag(111) and Cu(111). (a) the nonamer on Ag(111); (b) the claimed 18-mer on Cu(111); the inset is an image of the underlying Cu(111) lattice; (c) the 15-mer on Cu(111) from our study; d. the claimed nonamer on Cu(111); (e,f) nonamer and 11-mer on Ag(111) from our study. The length of black lines: 1.0 nm

in a and e; 1.6 nm in b, c, d, and f. hexamer, nonamer, and 10-mer on Ag(111) surface. The scale bar in c is 0.5 nm. Note: scale bars are added to the original images for a better comparison of the lateral size of the clusters. Figures are reproduced from the following references: (a,d), adapted with permission from[1] (copyright 2007 Springer Nature); (b) adapted with permission from[2] (copyright 2017 American Chemical Society).

Supplementary Discussion 1. Clusters on Ag(111)

We observed similar intermediate clusters and 15-mer's derivatives on Ag(111) exactly as that on both Pt(111) and Cu(111) that firstly the formation of hexagonal structures and then fused pentagonal structures. This observation strongly suggests that similar nucleation and growth processes hold for Ag(111) surface. 1, The formation of a hexagon ring; 2, the attachment of individual water molecules at ortho-positions along the central hexagon ring to form a tetragon; and 3, the formation of a pentagon with an additional water molecule bridged between the two molecules at the ortho-position. The pentagon rings could also be formed at the para-positions of the hexagon ring. Although the nucleation on Ag(111) shows more complex phenomena than that on Cu(111) and Pt(111), this observation suggests that the similar nucleation and growth processes can also occur on Ag(111) surface.

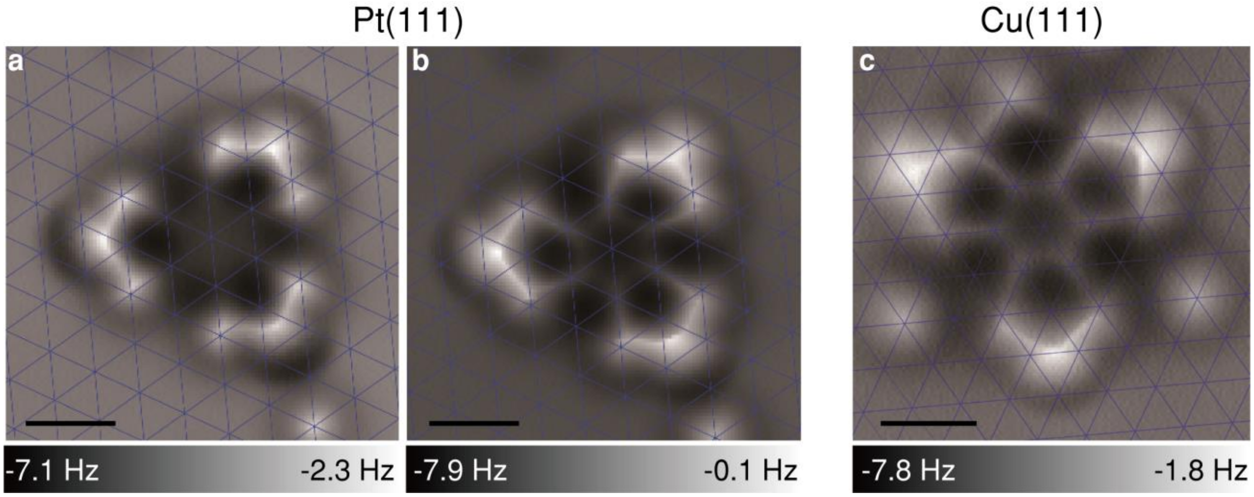

Supplementary Fig. 2 | Adsorption sites of 15-mer on Pt(111) and Cu(111). (a,b) Constant-height AFM images of water clusters on Pt(111) surface at different heights. The bright protrusion at the right bottom of a is a CO molecule and the CO is used to determine the top site of Pt. The adsorption sites in b are determined according to the adsorption sites in a. (c) The constant-height AFM images of water cluster on Cu(111) surface. The AFM image is acquired on another Cu(111) crystal. The three bright protrusions around the 15-mer are CO molecules. The determination of adsorption sites by AFM images are influenced by the distortion induced by CO tilt at close distance and the thermal drift. These factors may result in the differences in adsorption sites acquired by AFM images and calculations. The intersection points of the blue lines are the positions of Pt/Cu atoms. Scale bars: 0.5 nm.

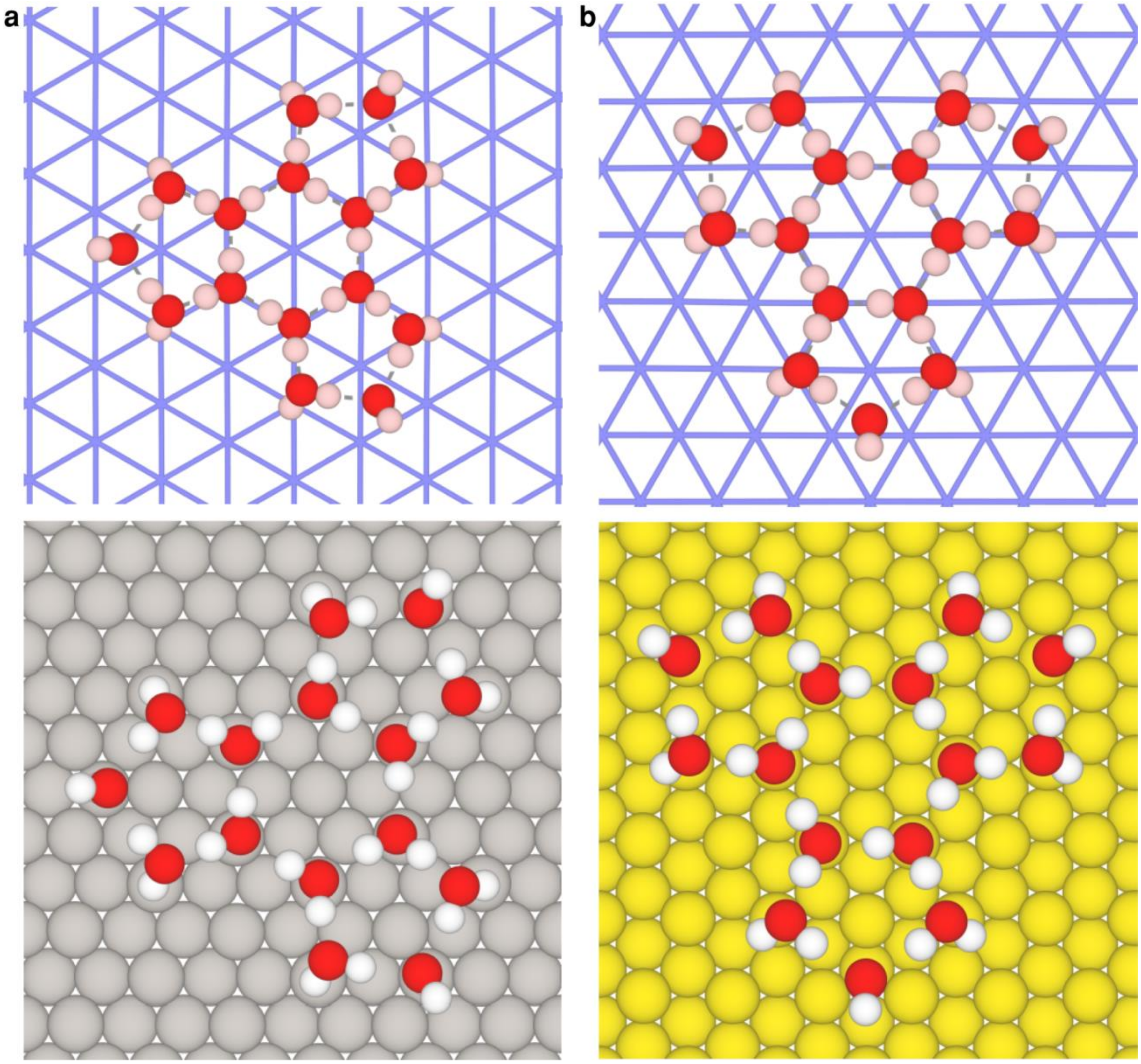

Supplementary Fig. 3 | Adsorption sites and top views of 15-mer calculated by DFT on (a) Pt(111) and (b) Cu(111). H and O atoms in the top panels are denoted as pink and red spheres, respectively gray. H and O atoms in the bottom panels are denoted as white and red spheres, respectively. Pt and Cu atoms are denoted as gray and yellow spheres, respectively. The intersection points of the blue lines are the positions of Pt/Cu atoms.

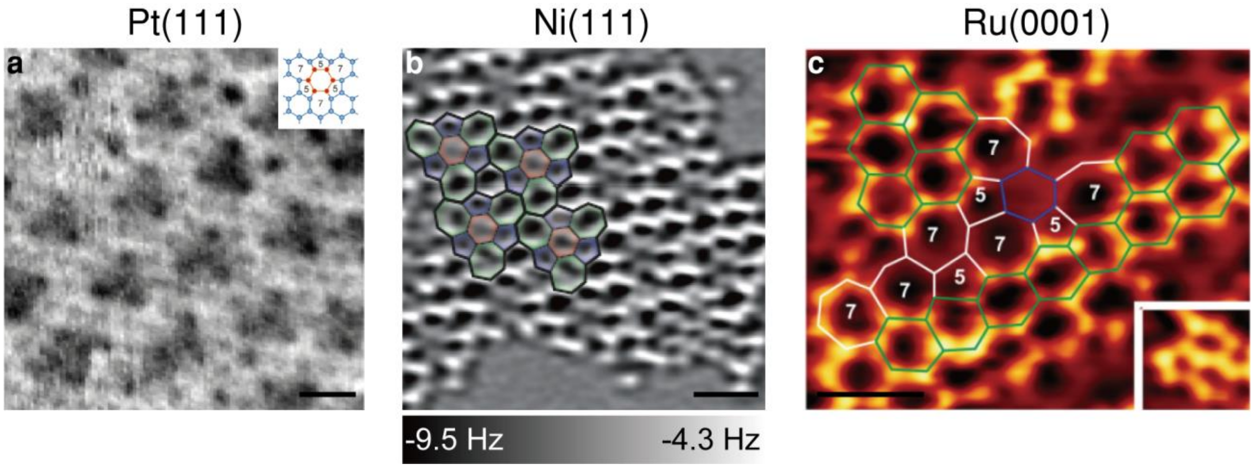

Supplementary Fig. 4 | STM images of 15-mer observed on hydrophilic metal surfaces. (a) 2D water network film on Pt(111). The blue and red flames indicate the pentagonal/heptagonal and hexagonal rings, respectively. (b) Water island on Ni(111). The blue, red, and green flames indicate the pentagonal, hexagonal, and heptagonal rings, respectively. (c) Water island on Ru(0001). The inset shows a pentagonal ring of water molecules. A water island with 15-mer is also observed on Pd(111)[5]. Scale bars: 1 nm. Figures are reproduced from the following references: a, adapted with permission from[3] (copyright 2010 American Physical Society); (b) adapted with permission from[4] (copyright 2019 American Physical Society); (c) adapted with permission from[5] (copyright 2012 American Physical Society).

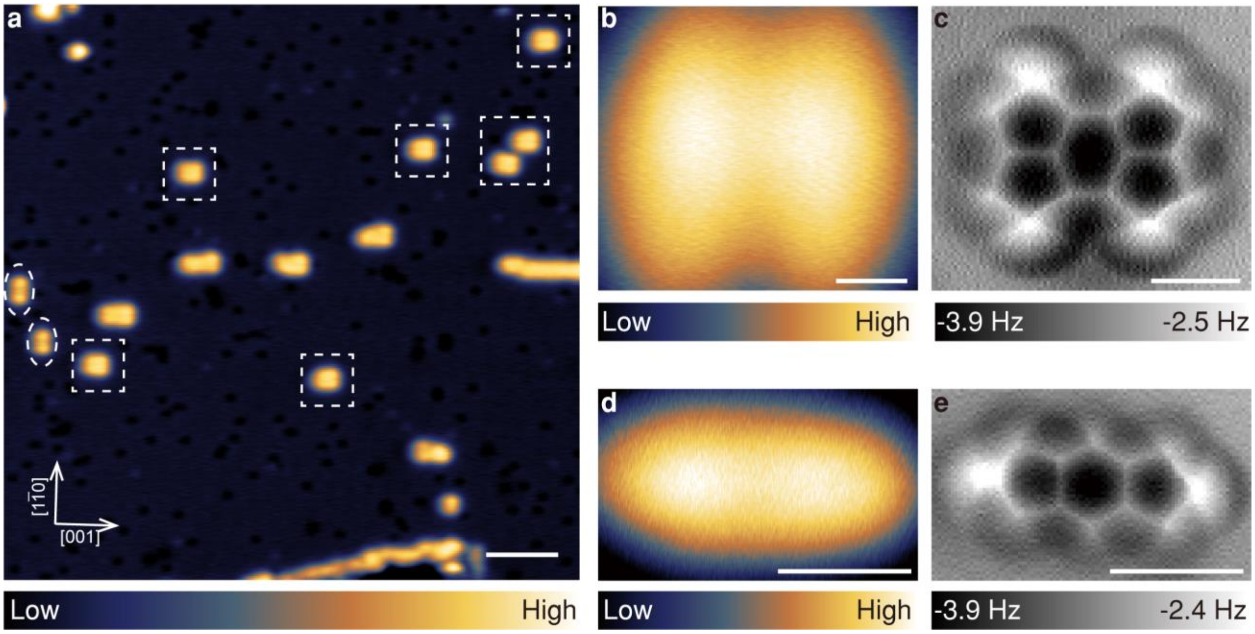

Supplementary Fig. 5 | SPM characterization of water clusters on Cu(110) grown at 78 K. (a) Constant-current STM image of water clusters on Cu(110) (V = 100 mV, I = 10 pA) with a metal tip. (b,d) Constant-current STM images of water clusters with two-fold symmetry on Cu(110) surface (V = 100 mV, I = 10 pA) with a metal tip. (c,e) The corresponding constant-height AFM images with a CO-terminated tip. The images in d and e are rotated 90° relative to a. The water clusters with two-fold symmetry are marked with white dashed squares and ellipses. The dark holes on the surface are CO molecules. Scale bar: 5 nm (a), 0.5 nm (b,c,d,e). It should be noted that Ref. 13 reported the same cluster as that of Supplementary Fig. 5b,c.

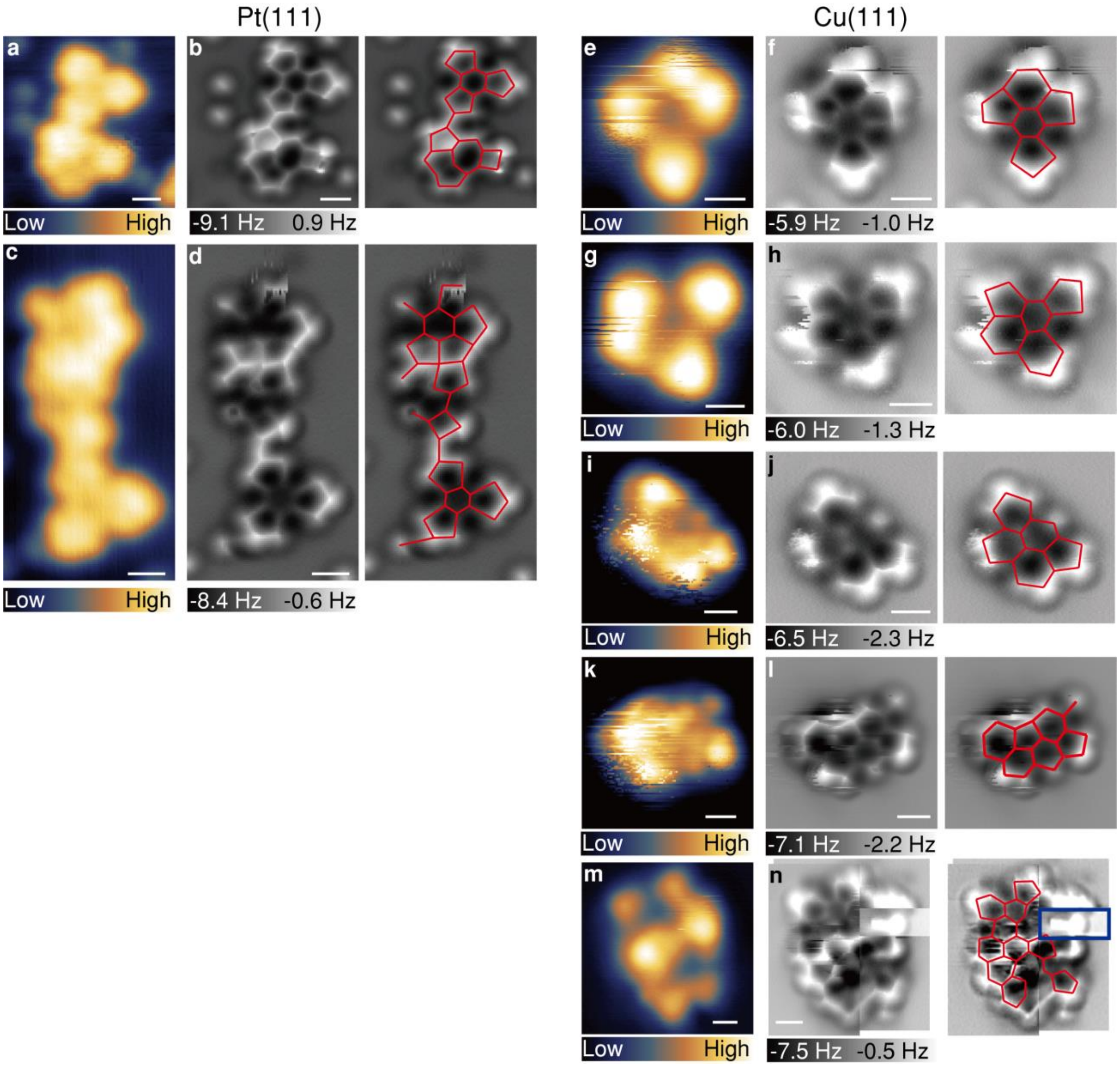

Supplementary Fig. 6 | SPM characterization of large water clusters on Pt(111) and Cu(111) with a CO-terminated tip. (a,c) Constant-current STM images (V = 100 mV, I = 30 pA) of water clusters on Pt(111) surface. (b,d) The corresponding constant-height AFM images. (e,g,i,k,m) Constant-current STM images of water clusters on Cu(111) surface. (f,h,j,l,n) The corresponding constant-height AFM images. The clusters in f, h, and j consist of 17, 18, and 18 water molecules and have several hexagonal rings. The cluster in n has water molecules at the second layer. To image the structure of the first layer, the AFM images are matched by two AFM images at different tip heights. For the part of AFM image at the right marked by blue rectangle, the tip height is 120 pm higher than the other parts. Scale bars: 0.5 nm.

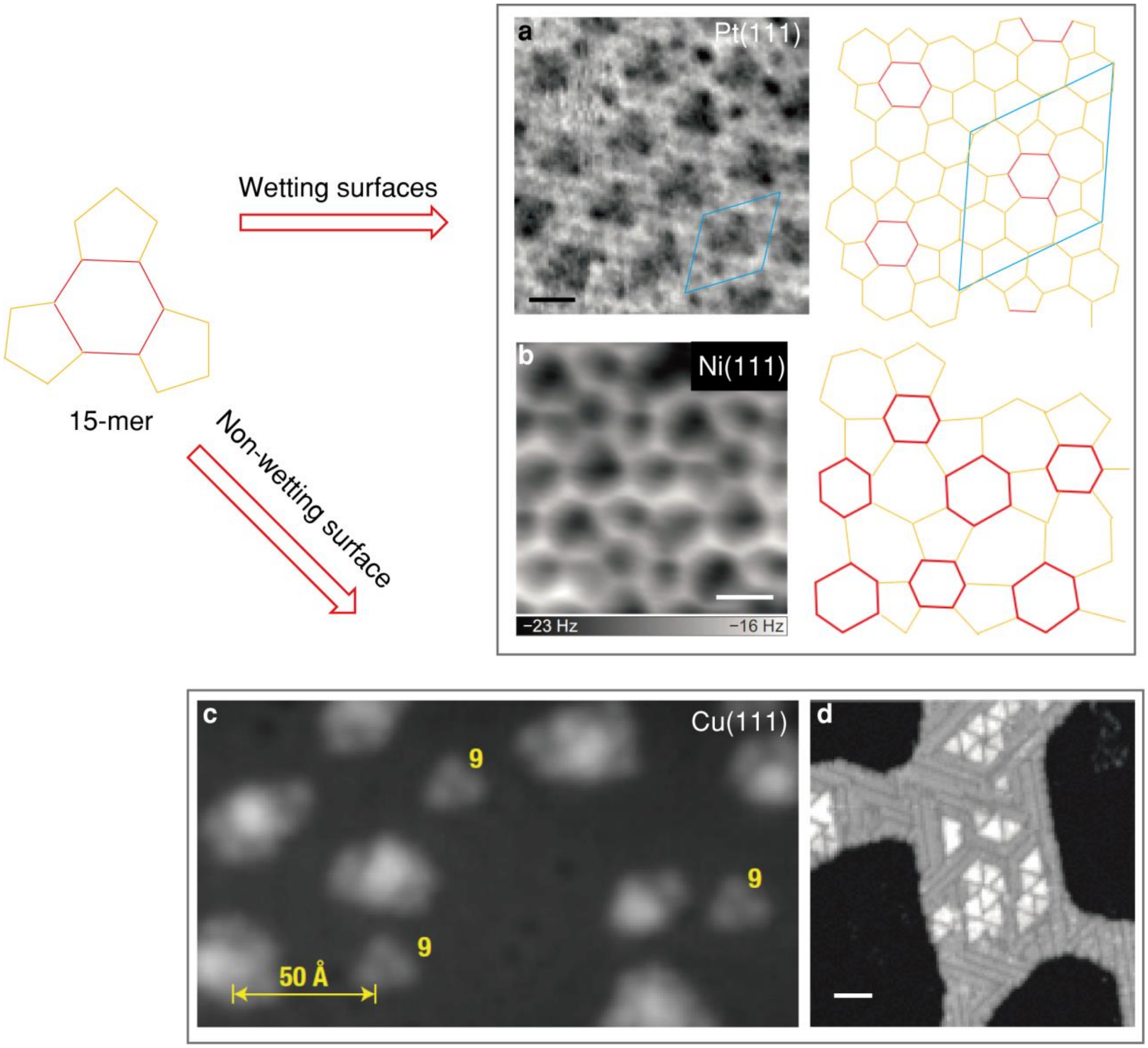

Supplementary Fig. 7 | Evolution from 15-mer to 2D film (3D island) on wetting (non-wetting) surfaces. (a) 2D water network film on Pt(111) (scale bar: 1.0 nm). (b) Constant height AFM image of water island on Ni(111) (scale bar: 0.5 nm) and corresponding structure. (c) Water clusters on Cu(111). (d) Pyramids and crystalline 3D island on Cu(111) (scale bar: 10 nm). Figures are reproduced from the following references: (a) adapted with permission from[3] (copyright 2010 American Physical Society); (b) adapted with permission from[4] (copyright 2019 American Physical Society); (c) adapted with permission from[1] (copyright 2007 Springer Nature); (d) adapted with permission from[6] (copyright 2007 American Physical Society).

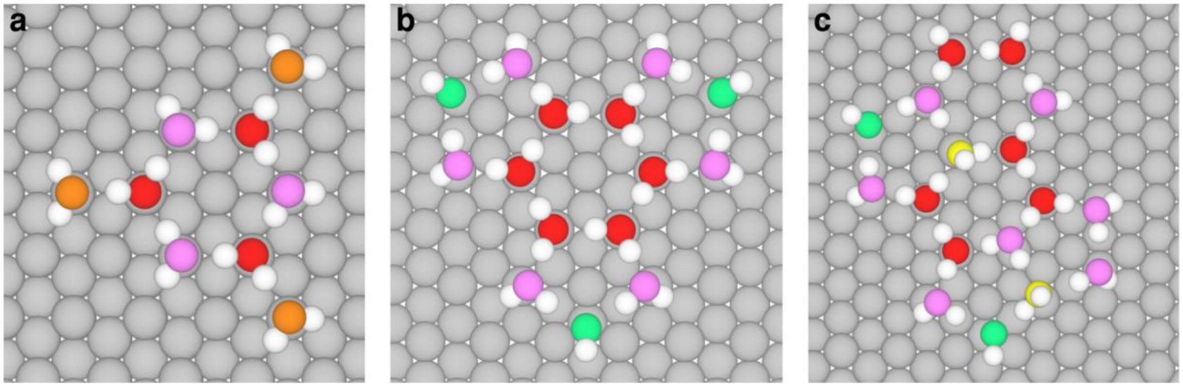

Supplementary Fig. 8 | Three representative clusters which contain different kind of water molecules sorted in Table 1. (a-c) Nonamer, 15-mer, and 17-mer on Pt(111) surface. The H atoms are denoted as white spheres. The O atoms in Low water molecules in a are denoted as red (connected with Branch water) and purple (not connected with Branch water) spheres, respectively. The O atoms in Branch water molecules in a are denoted as brown spheres. The O atoms in Low, High, H-down, and H-up water molecules in b and c are denoted as red, pink, green, and yellow spheres, respectively. Pt atoms are denoted as gray spheres.

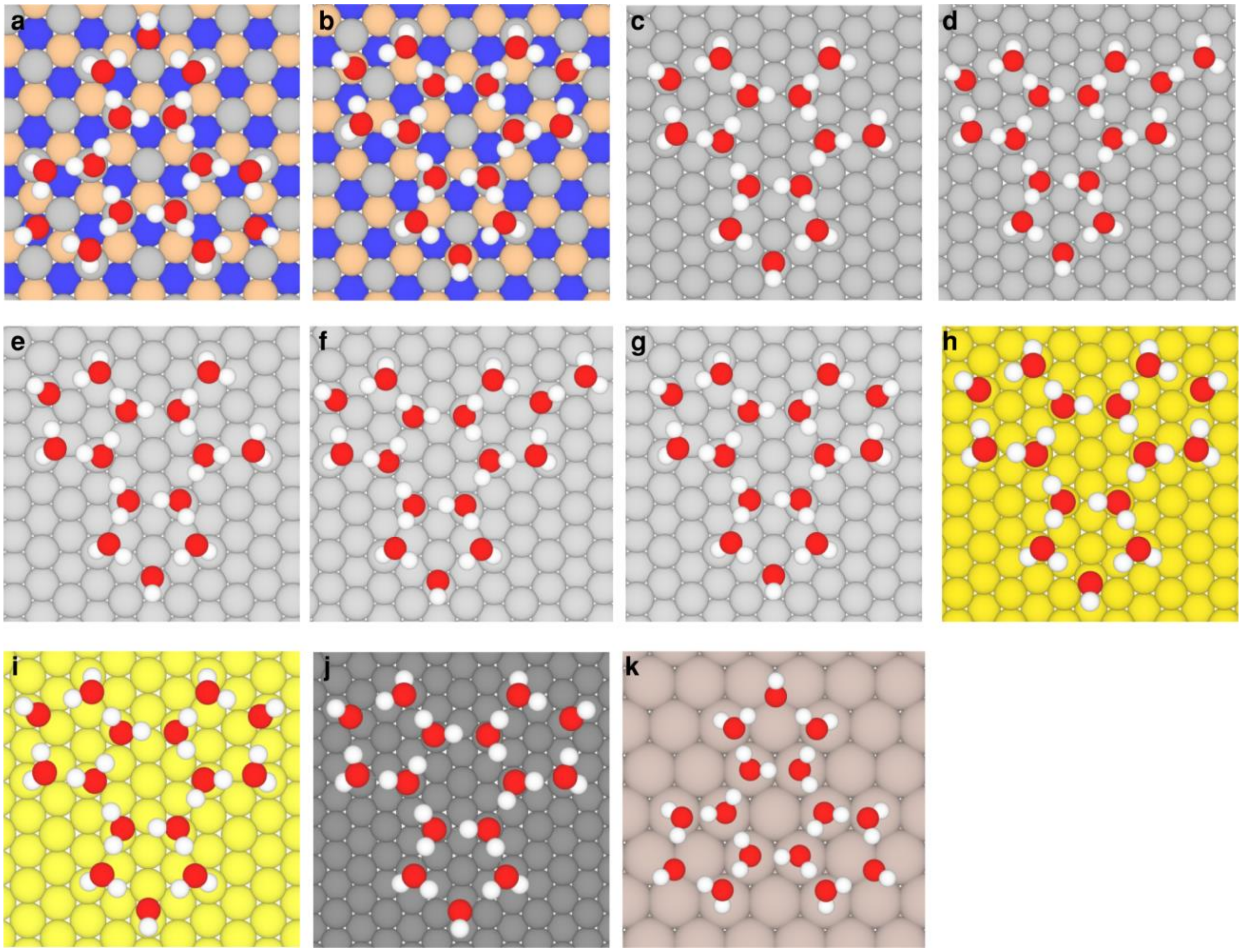

Supplementary Fig. 9 | The top view of water clusters used for DFT calculations is in Supplementary Table 1. (a,b) Schematic diagrams for 15-mers absorbed on fcc hollow sites or hcp hollow sites of Pt(111). The first layer, second layer and third layer of substrates are drawn as grey, orange, and blue, respectively. (c,d) 14-mer and 16-mer on Pt(111) surface. (e,f) 14-mer and 16-mer on Ag(111) surface. (g-k) 15-mer on Ag(111), Cu(111), Au(111), Ni(111), and Ru(0001) surface. The H and O atoms are denoted as white and red spheres, respectively.

Supplementary references

1. Michaelides, A. & Morgenstern, K. Ice nanoclusters at hydrophobic metal surfaces. *Nat. Mater.* **6**, 597–601 (2007).

2. Liriano, M. L. et al. Water–ice analogues of polycyclic aromatic hydrocarbons: water nanoclusters on Cu(111). *J. Am. Chem. Soc.* **139**, 6403–6410 (2017).

3. Nie, S., Feibelman, P. J., Bartelt, N. C. & Thürmer, K. Pentagons and heptagons in the first water layer on Pt(111). *Phys. Rev. Lett.* **105**, 026102 (2010).

4. Shiotari, A., Sugimoto, Y. & Kamio, H. Characterization of two- and one-dimensional water networks on Ni(111) via atomic force microscopy. *Phys. Rev. Mater.* **3**, 093001 (2019).

5. Maier, S. et al. Adsorbed water-molecule hexagons with unexpected rotations in islands on Ru(0001) and Pd(111). *Phys. Rev. B* **85**, 155434 (2012).

6. Mehlhorn, M. & Morgenstern, K. Faceting during the transformation of amorphous to crystalline ice. *Phys. Rev. Lett.* **99**, 246101 (2007).
